# Hypobaric hypoxia preconditioning protects against hypothalamic neuron apoptosis in heat-exposed rats by reversing hypothalamic overexpression of matrix metalloproteinase-9 and ischemia

**DOI:** 10.7150/ijms.47560

**Published:** 2020-09-20

**Authors:** Chien-Ming Chao, Chun-Liang Chen, Ko-Chi Niu, Cheng-Hsien Lin, Ling-Yu Tang, Lieh-Sheng Lin, Ching-Ping Chang

**Affiliations:** 1 Department of Intensive Care Medicine, Chi Mei Medical Center, Liouying, Tainan, Taiwan.; 2 Department of Nursing, Min-Hwei College of Health Care Management, Tainan, Taiwan.; 3 Department of Gastroenterology and General Surgery, Chi Mei Medical Hospital, Chiali, Tainan, Taiwan.; 4 Department of Hyperbaric Oxygen, Chi Mei Medical Center, Tainan, Taiwan.; 5 Department of Medicine, Mackay Medical College, New Taipei City, Taiwan.; 6 Department of Medical Research, Chi Mei Medical Center, Tainan, Taiwan.; 7 Department of Neurosurgery, Taipei Medical University Hospital, Taipei, Taiwan.

**Keywords:** hypothalamus, ischemic/hypoxic injury, heatstroke, hypobaric hypoxia

## Abstract

**Background:** Hypoxia-inducible factor-1α (HIF-1α), heat shock protein-72 (HSP-72), hemeoxygenase-1 (HO-1), and matrix metalloproteinase-9 (MMP-9) have been identified as potential therapeutic targets in the brain for cerebral ischemia. To elucidate their underlying mechanisms, we first aimed to ascertain whether these proteins participate in the pathogenesis of heat-induced ischemic damage to the hypothalamus of rats. Second, we investigated whether hypobaric hypoxia preconditioning (HHP) attenuates heat-induced hypothalamic ischemic/hypoxic injury by modulating these proteins *in situ*.

**Methods:** Anesthetized rats treated with or without HHP were subjected to heat stress. Hypothalamic ischemic/hypoxic damage was evaluated by measuring hypothalamic levels of cerebral blood flow (CBF), partial oxygen pressure (PO_2_), and hypothalamic temperature via an implanted probe. Hypothalamic apoptotic neurons were counted by measuring the number of NeuN/caspase-3/DAPI triple-stained cells. Hypothalamic protein expression of HIF-1α, HSP-72, HO-1, and MMP-9 was determined biochemically.

**Results:** Before the start of the thermal experiments, rats were subjected to 5 hours of HHP (0.66 ATA or 18.3% O_2_) daily for 5 consecutive days per week for 2 weeks, which led to significant loss of body weight, reduced brown adipose tissue (BAT) wet weight and decreased body temperature. The animals were then subjected to thermal studies. Twenty minutes after heat stress, heat-exposed rats not treated with HHP displayed significantly higher core and hypothalamic temperatures, hypothalamic MMP-9 levels, and numbers of hypothalamic apoptotic neurons but significantly lower mean blood pressure, hypothalamic blood flow, and PO_2_ values than control rats not exposed to heat. In heat-exposed rats, HHP significantly increased the hypothalamic levels of HIF-1α, HSP-72, and HO-1 but significantly alleviated body and hypothalamic hyperthermia, hypotension, hypothalamic ischemia, hypoxia, neuronal apoptosis and degeneration.

**Conclusions:** HHP may protect against hypothalamic ischemic/hypoxic injury and overexpression of MMP-9 by upregulating the hypothalamic expression of HIF-1α, HSP-72, and HO-1 in rats subjected to heatstroke.

## Introduction

Heatstroke is characterized by hyperthermia, systemic inflammation, and multiple organ dysfunction syndromes, including arterial hypotension 1-3. In particular, arterial hypotension and subsequent hypothalamic ischemic injury may be the main causes of heatstroke 1, 4.

In rodents, heat-induced thermoregulatory deficits and mortality may result from ischemic and oxidative damage to the hypothalamus (the essential thermoregulatory center in the mammalian brain). Compared to normothermic controls, mice subjected to heatstroke exhibit significantly higher levels of cellular ischemia markers (e.g., glutamate and the lactate-to-pyruvate ratio) and damage markers (e.g., glycerol) in the hypothalamus 5, 6. In cerebral ischemia, enhanced expression of matrix metalloproteinase-9 (MMP-9) is associated with various complications, including neuronal damage, apoptosis, and blood-brain barrier (BBB) disruption 7. Hemeoxygenase-1 (HO-1) protects cells and tissues by exerting anti-inflammatory, antiapoptotic, antiproliferative, and antioxidative effects in response to noxious stimuli 8. Hypoxia-inducible factor-1α (HIF-1α) acts as a potential therapeutic target for cerebral ischemia 9. Heat shock protein-72 (HSP-72) overexpression appears to be critical for the development of thermotolerance and protection against heat-induced hypothalamic ischemic and oxidative damage in rodents. However, the interrelationships among these proteins in the hypothalamus during the pathogenesis of heatstroke remain unclear.

Changes associated with acclimation to hypoxia, including carotid body-mediated minute ventilation, which can improve oxygen saturation 10, diuresis, which can effectively increase hemoglobin and hematocrit concentrations, and stimulation of erythropoiesis, which can increase the number and oxygen-carrying capacity of red blood cells; occur within 1-3 weeks of high-altitude living (1500-3000 meters) or intermittent exposure to hypoxia 11. These systemic responses have been experimentally linked to several downstream effects of HIF-1α, HSP-72 and other related proteins in hypothalamic tissues.

Hypobaric hypoxia preconditioning (HHP) is a transient phenomenon caused by brief episodes of sublethal hypoxia that induce various endogenous atrophic signals and robustly protect against subsequent lethal insults 12, 13. Although HHP has been shown to exert neuroprotective effects against ischemic brain injury 13, 14, it is still unknown whether HHP protects against heat-induced hypothalamic injury 15 by modulating hypothalamic proteins including HIF-α, MMP-9, HSP-72, and HO-1.

To address this question, the present study aimed to assess the effects of HHP on heat-induced ischemic and hypoxic damage to the rat hypothalamus. In addition, the temporal profiles of HIF-1α, HSP-72, HO-1, and MMP-9 in the hypothalamus were assessed in rats subjected to heatstroke and treated with or without HHP.

## Materials and Methods

### Animal care

All animal experiments were conducted on adult male Sprague-Dawley rats weighing 248-306 g. All experimental and animal care protocols were approved by the Institutional Animal Care and Use Committee of Chi Mei Medical Center, Tainan, Taiwan (approval no.: 106121110) in accordance with the guidelines of Guide for the Care and Use of Laboratory Animals published by the US National Institutes of Health 16, and care was taken to minimize pain and suffering. The rats were housed in a temperature-controlled room (24±1.0 °C) with a relative humidity of 50±10% on a 12/12 hour-light/dark cycle, and food pellets and water were provided *ad libitum*.

### Physiological variable monitoring

Each animal was anesthetized with sodium pentobarbital (40 mg/kg body weight, i.p.), and the femoral artery was cannulated with polyethylene tubing to measure mean arterial blood pressure (MABP) and heart rate (HR). Body core temperature (Tco) was monitored continuously by a thermocouple inserted into the rectum, and MABP and HR were continuously monitored with a pressure transducer. The head of the animal was mounted on a stereotaxic apparatus (David Kopf Instruments, Tujunga, CA, USA) with the nose bar positioned 3.3 mm below the horizontal line. Following a midline incision, the skull was exposed, and a burr hole was made in the skull to allow insertion of a 100-μm-diameter thermocouple and two 230-μm fibers attached to an oxygen probe. This combined probe was used to measure oxygen, temperature, and microvascular blood flow. OxyLite and OxyFlo instruments were used for the measurements. The OxyLite 2000 instrument (Oxford Optronix, Oxford, UK) is a two-channel device (measures partial oxygen pressure [PO_2_] and temperature at 2 locations simultaneously), whereas the OxyFlo 2000 instrument is a two-channel laser Doppler perfusion monitoring instrument. Together, these 2 instruments simultaneously provided tissue blood flow, oxygenation, and temperature data 17.

### HHP simulation

Animals in the HHP groups were exposed to 5 hours of simulated HHP of 4000 m in a hypobaric chamber (0.66 ATA or 18.3% O_2_) daily for 5 consecutive days per week for 2 weeks before the start of heat exposure 18. All animals in the non-HPP groups were exposed to 1.0 ATA (21% O_2_) for the equivalent period of time.

### Induction of heatstroke

The Tco of the normothermic control group was maintained at about 37 °C with a folded heating pad at a room temperature of 26 °C throughout the entire experiment. For the heatstroke group, the heating pad was removed before the start of heat stress (ambient temperature [Ta] of 42 °C with a relative humidity of 60%). In the heatstroke group, an MABP value of 25 mmHg below the peak accompanied by excessive body hyperthermia (i.e., ~41.5 °C Tco and ~40.0 °C hypothalamic temperature) was observed approximately 70 min after the initiation of heat stress; this time point was arbitrarily used as the onset of heatstroke 19. Heat was withdrawn from the heat-exposed animals 70 min after heat stress, and the animals were allowed to recover at room temperature (26 °C).

### Experimental groups

The animals were randomly assigned randomly to one of the following four groups: the non-HHP + non-heat-exposed group, HHP + non-heat-exposed group, non-HP + heat-exposed group, and HHP + heat-exposed group. In the non-heat-exposed groups, the Tco was maintained at approximately 37 °C with a heating pad. The animals in the heat-exposed groups were exposed to heat stress (40 °C) for exactly 70 min.

### Western blot analysis

The rats were anesthetized and underwent intracardiac perfusion with 0.1 M PBS (pH 7.4) 20, 21. The hypothalamic region of the brain was rapidly isolated, and the brain tissues were homogenized using a homogenizer. Total protein was extracted using protein extraction reagent (Bio-Rad Laboratories, CA, USA), and the protein concentration was quantified by using the Bradford method (Bio-Rad protein assay kit, Bio-Rad Laboratories). The protein samples (50 μg) were separated by 20% sodium dodecyl sulfate-polyacrylamide gel electrophoresis and were subsequently transferred onto polyvinylidene fluoride membranes. The blots were blocked and incubated with a primary antibody specific for HSP-72, HIF-1α or HO-1. The membranes were then incubated with horseradish peroxidase-conjugated anti-rabbit immunoglobulin G and anti-mouse IgG. Following secondary antibody incubation, the immunoblots were visualized with an enhanced chemiluminescence detection system. The expression of HSP-72, HIF-1α, HO-1 and β-actin was semiquantified using a gel densitometry scanning program.

### Gelatin zymography

The levels of MMP-9 in the brain homogenates were measured by gelatin zymogram following a previously described technique 22. The brains were quickly removed from the rats under general anesthesia, and the tissues were frozen immediately in liquid nitrogen and stored at -80 °C. Total protein concentrations were determined using the bicinchoninic acid protein assay. Protein samples (50 μg) were prepared, loaded on 10% tris-glycine gels with 0.1% gelatin as a substrate and separated. After electrophoresis and incubation, the gels were stained with 3% methanol, 10% acetic acid, and 0.5% wt/vol Coomassie brilliant blue for 2 hours and then destained. Gelatinolytic activity was manifested as horizontal white bands on a blue background. MMP bands were identified after standard techniques using molecular weight criteria and a human MMP-9 control, which was purchased from Chemicon 23.

### Hematoxylin and Eosin (H&E) staining

We evaluated brain tissue damage following a previously described method 24. At the end of the experiments, the rats were anesthetized as described above and intracardially perfused with isotonic chloride solution followed by 4% (w/v) paraformaldehyde (Bio-Rad Laboratories) in 0.1 M sodium phosphate buffer (pH 7.4; Bio-Rad Laboratories). The brains were subsequently removed, fixed for 48 hours in 4% (w/v) paraformaldehyde, and embedded in paraffin (Bio-Rad Laboratories), and brain tissues were sliced into 4-μm coronal sections and stained with hematoxylin-eosin (H&E) (Sigma-Aldrich, MA, USA).

Semiquantitative histological analysis was performed after H&E staining to evaluate morphological changes in neural cells and the severity of damage to the brain following heat stress. Brain sections (corresponding to coronal coordinates of Paxinos and Watson 25 at bregma -2.2~-3.6 mm, lateral 0~1 mm, and depth 8.0~10 mm) were semiquantitatively scored 26 from 0 to 4 (0= no detectable lesion, 1 = lesion involving <25% of the field, 2 = lesion involving 25%-50% of the field, 3 = lesion involving 50%-75% of the field, and 4 = lesion involving >75% of the field). Each hypothalamus region was evaluated for morphological changes as follows 27: 0 = normal morphology, 1 =minor damage (edema and few pyknotic cells), 2 = moderate damage (structural disorganization, edema, many pyknotic cells, and vacuolization), and 3 = intense damage (structural disorganization, edema, many pyknotic cells, and vacuolization). Brain damage was scored and evaluated, and the scores were multiplied to yield final scores, which ranged from 0 to 12, for each animal. Representative images were collected.

### Neuronal apoptosis

Coronal sections were incubated with 10% normal donkey serum, primary antibodies (rabbit anti-rat neuron-specific nuclear protein [NeuN; 1:200, Santa Cruz Biotechnology] and rabbit anti-cleaved caspase-3 [1:100; Santa Cruz Biotechnology]) and fluorescent-labeled secondary antibodies (Alexa Fluor 568-conjugated goat anti-rabbit IgG, Alexa Fluor 568-conjugated goat anti-mouse IgG or Alexa Fluor 488-conjugated goat anti-mouse; Santa Cruz Biotechnology). The sections were incubated with 4,6-diamidino-2-phenylindole (DAPI, Vectashield ®Vector Laboratories, Burlingame, CA, USA) to counterstain the nuclei. Another section was stained with a terminal deoxyribonucleotide transferase-mediated dUTP nick end labeling (TUNEL) assay kit (#630108, Clontech, Palo Alto, CA, USA) to quantify neuronal apoptosis. The sections were subsequently washed with phosphate buffer, and the nuclei were counterstained with DAPI.

### Determination of hypothalamic glutamate levels, glycerol levels and lactate-to-pyruvate ratio

To measure hypothalamic glutamate concentrations, glycerol concentrations, and lactate-to-pyruvate ratio, aliquots of samples were injected into a CMA600 microdialysis analyzer (Carnegie Medicine, Stockholm, Sweden) 28.

### Data presentation and statistical analysis

The data are presented as the means±SDs ANOVA was used to analyze factorial experiments, and Duncan's multiple range test was used for post hoc comparisons among means. GraphPad Prism (version 7.01 for Windows; GraphPad Software, San Diego, CA, USA) was used for these analyses. A *p*-value of less than 0.05 was considered statistically significant.

## Results

### HHP reduces body weight, brown adipose tissue (BAT) weight and temperature

As shown in Table 1, after 2 weeks, the average body weight of HHP-treated rats was significantly lower than that of untreated rats, which remained unchanged. Both the BAT temperature and weight values of the HHP group were significantly lower than those of the non-HHP group (Table 1).

### HHP attenuates heat-induced hyperthermia, hypotension and hypothalamic ischemia/hypoxia

**Figure 1:**
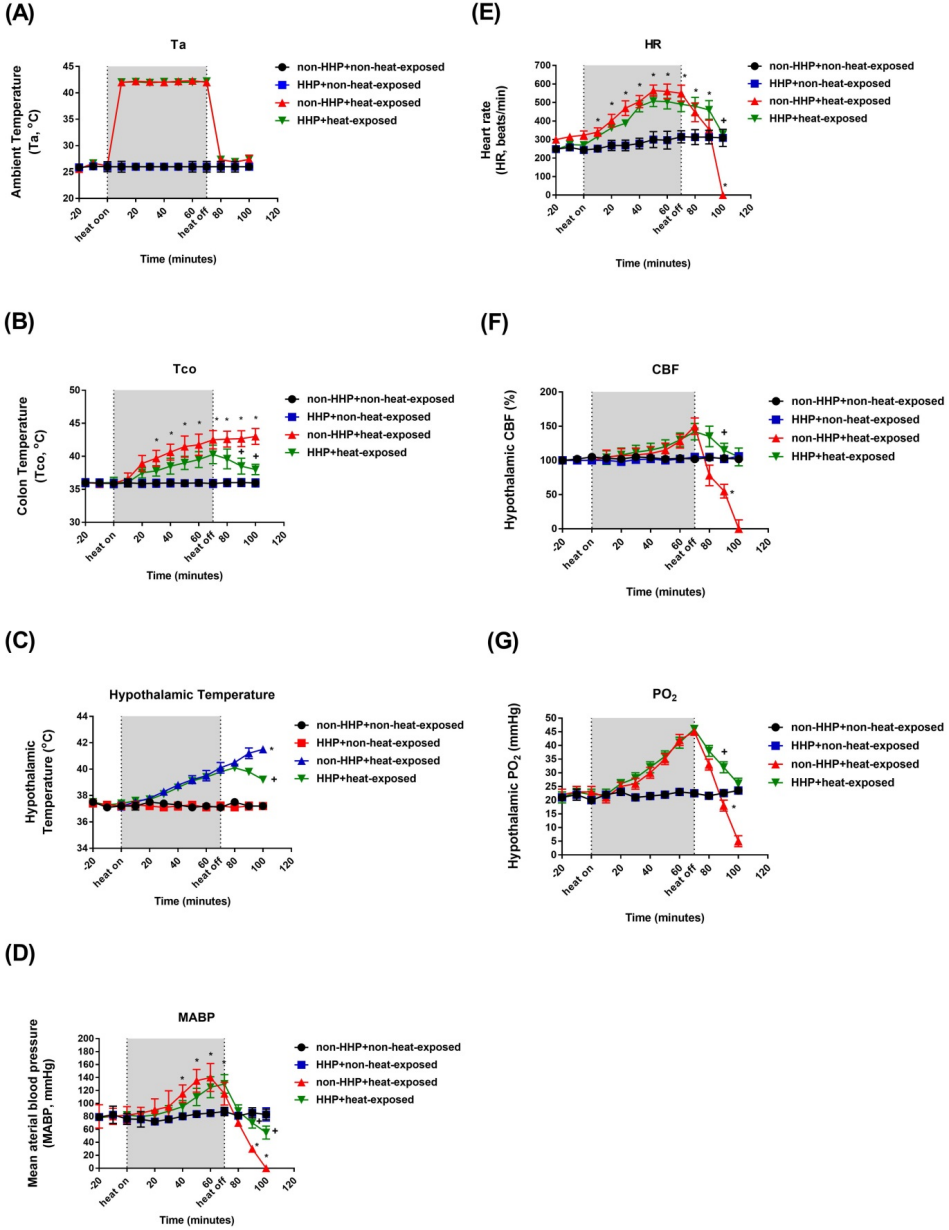
Changes in Ta (A), Tco (B), MABP (C), HR (D), hypothalamic temperature (E), CBF (F) and PO_2_ (G) in non-HHP + non-heat-exposed rats (⚫), HHP + non-heat-exposed rats (■), non-HHP + heat-exposed rats (▲) and HHP + heat-exposed rats (▼) over time. The values are the means±SDs; n=10 animals per group. *P<0.05 vs non-HHP + non-heat-exposed rats; +*P*<0.05 vs non-HHP + heat-exposed rats. Twenty minutes after heat exposure concluded, the heat-exposed rats not treated with HHP (non-HHP) displayed hyperthermia (~42 °C vs. ~37 °C), hypotension (~20 mmHg vs. ~90 mmHg), bradycardia (~300 beats/min vs. ~450 beats/min), hypothalamic ischemia (~50% vs. ~100%), and hypothalamic hypoxia (~15 mmHg vs. 22 mmHg).

**Figure 2:**
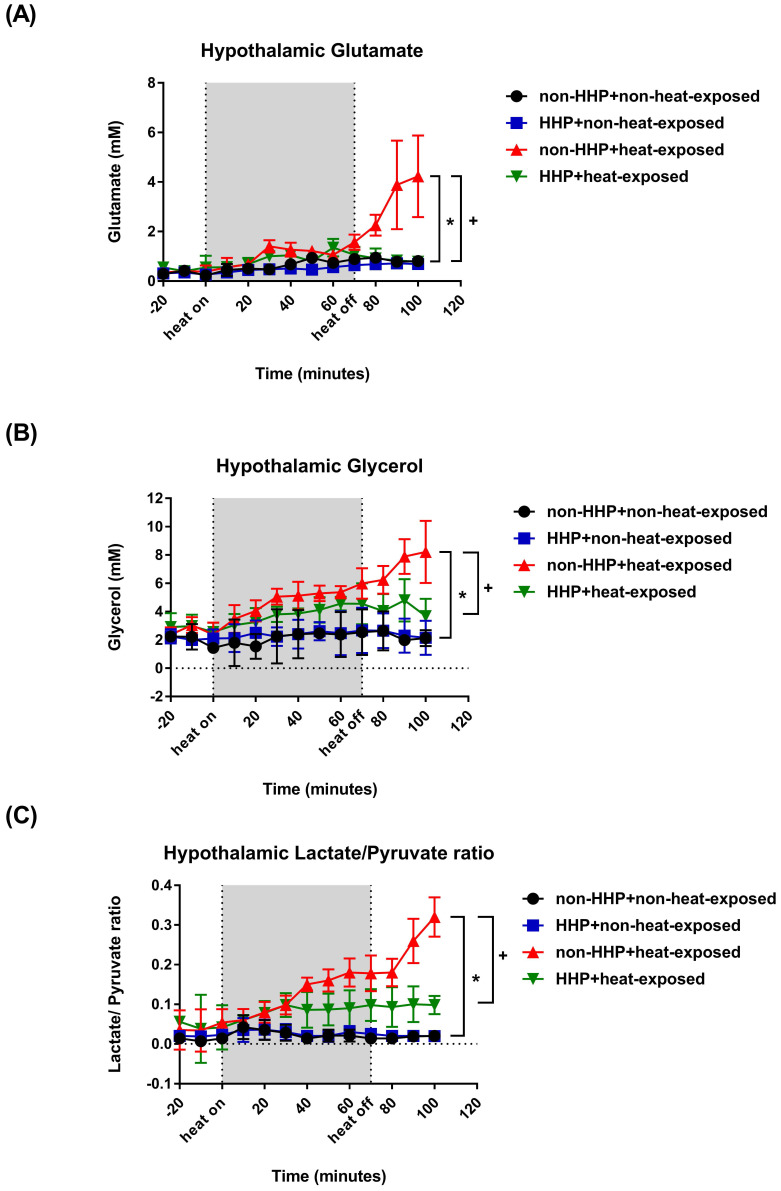
Changes in hypothalamic (A) glutamate levels, (B) glycerol levels, and (C) lactate/pyruvate ratio in non-HHP+non-heat-exposed rats (⚫), HHP + non-heat-exposed rats (■), non-HHP + heat-exposed rats (▲) and HHP + heat-exposed rats (▼) over time. The values are the means± SDs; n=10 rats per group. *P<0.05 vs. non-HHP+non-heat-exposed group; +*P*<0.05 vs. non-HHP+heat-exposed group.

Compared to the non-HHP + non-heat-exposed group, the non-HHP + heat-exposed rats exhibited significant increases in Tco (Fig. 1B; ~42 °C vs. ~37 °C) and hypothalamic temperature (Fig. 1C; ~41 °C vs. ~37 °C) but significant decreases in MABP (Fig. 1 D; ~20 mmHg vs. ~90 mmHg), HR (Fig. 1E; ~300 beats/min vs. ~450 beats/min), hypothalamic cerebral blood flow (CBF) (Fig. 1F; ~50% vs. ~100%), and hypothalamic PO_2_ (Fig. 1G; ~15 mmHg vs. ~22 mmHg) 20 min after heatstroke (or 90 min after the start of 70 min of heat stress; Fig. 1A). Heat-induced hypotension and hypothalamic ischemia/hypoxia were significantly attenuated by HHP, as shown in the HHP + heat-exposed rats (MABP, ~80 mmHg; CBF, ~120%; PO_2_, ~30 mmHg). Biochemical assays also confirmed that cellular ischemia indicators, namely, glutamate levels and the lactate/pyruvate ratio, and a cellular damage indicator, namely, glycerol levels (Fig. 2), were significantly increased in the hypothalamus by heat stress. HHP significantly attenuated the increases in these cellular ischemia and injury indicators in the hypothalamus (Fig. 2).

### HHP reduces heat-induced increases in hypothalamic cell damage scores and the number of hypothalamic apoptotic neurons

**Figure 3:**
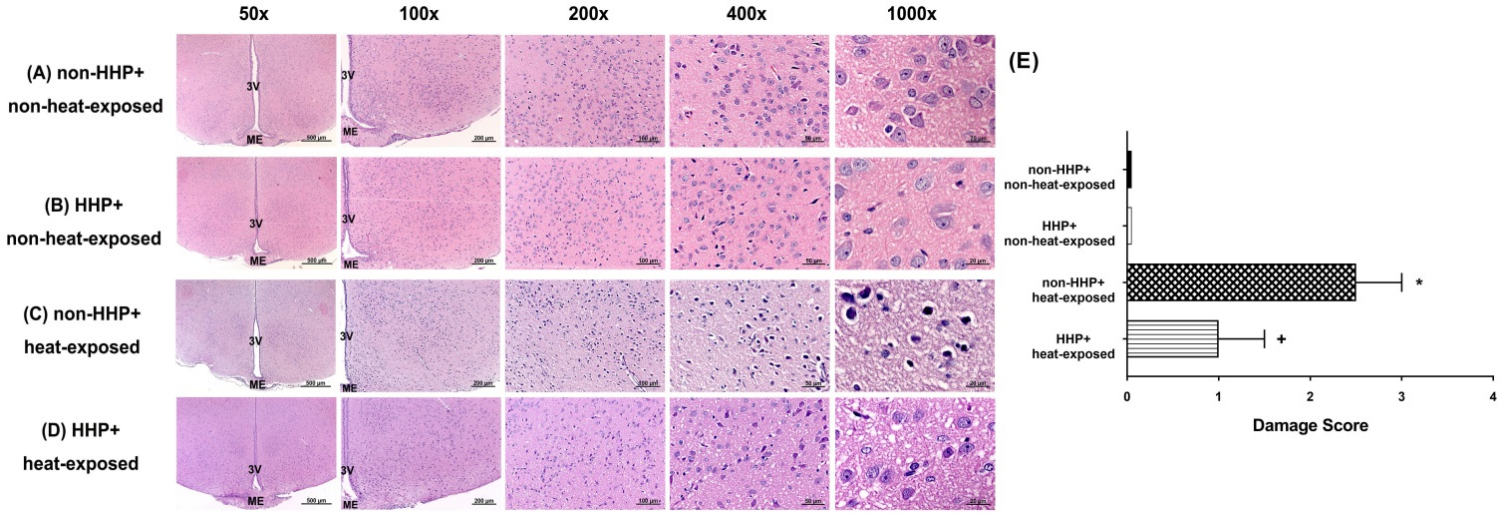
(A) Photomicrographs of hypothalamic hematoxylin-eosin staining of a non-HHP + non-heat-exposed rat (A), an HHP + non-heat-exposed rat (B), a non-HHP + heat-exposed rat (C), and an HHP + heat-exposed rat (D). Images of brain morphology are shown at 50x, 100x, 200x, 400x and 1000x magnification. Scale bars= 500 μm, 200 μm, 100 μm, 50 μm, and 20 μm. Rats in the heat-exposed groups were killed 90 min after heat stress, and rats in the non-heat-exposed groups were skilled at the equivalent time. (E) The hypothalamic damage scores of the different groups are presented as the means±SDs; n=10 per group. *P<0.05 for the non-HHP + heat-exposed group versus the non-HHP + non-heat-exposed group; ^+^*P*<0.05 for the HHP + heat-exposed group versus the non-HHP + heat-exposed group. 3V= third ventricle; ME= median eminence.

**Figure 4:**
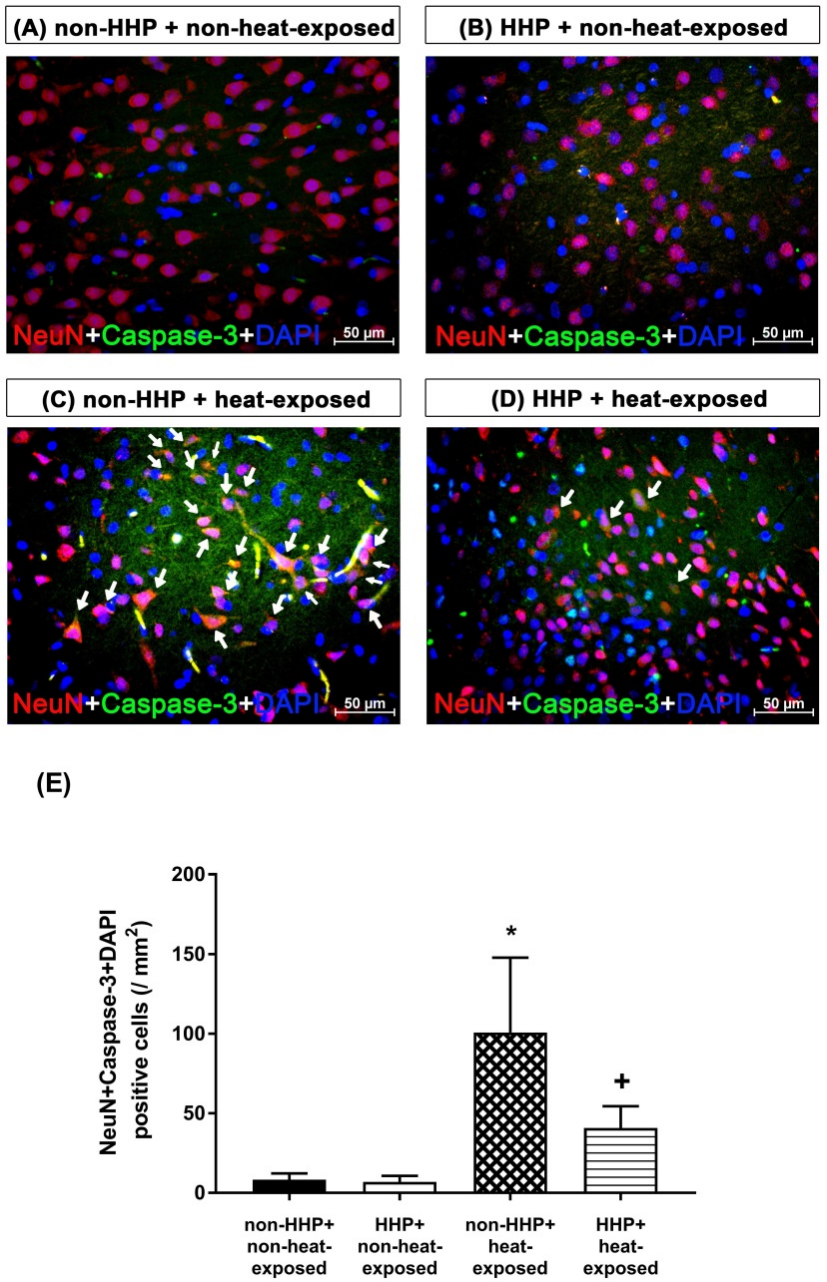
Photomicrographs of immunofluorescence staining of hypothalamic apoptotic neurons (NeuN+caspase-3+DAPI-positive cells) in a non-HHP + non-heat-exposed rat (A), an HHP + non-heat-exposed rat (B), a non-HHP+heat-exposed rat (C), and an HHP+heat-exposed rat (D). The animals were killed 90 min after heat stress or at the equivalent time for the non-heat-exposed groups. Scale bar=50 µm. (E) The values are presented as the means±SDs; n=10 per group. *P<0.05 for the non-HHP + heat-exposed group versus the non-HHP + non-heat-exposed group; ^+^*P*<0.05 for the HHP + heat-exposed group versus the non-HHP + heat-exposed group.

**Figure 5:**
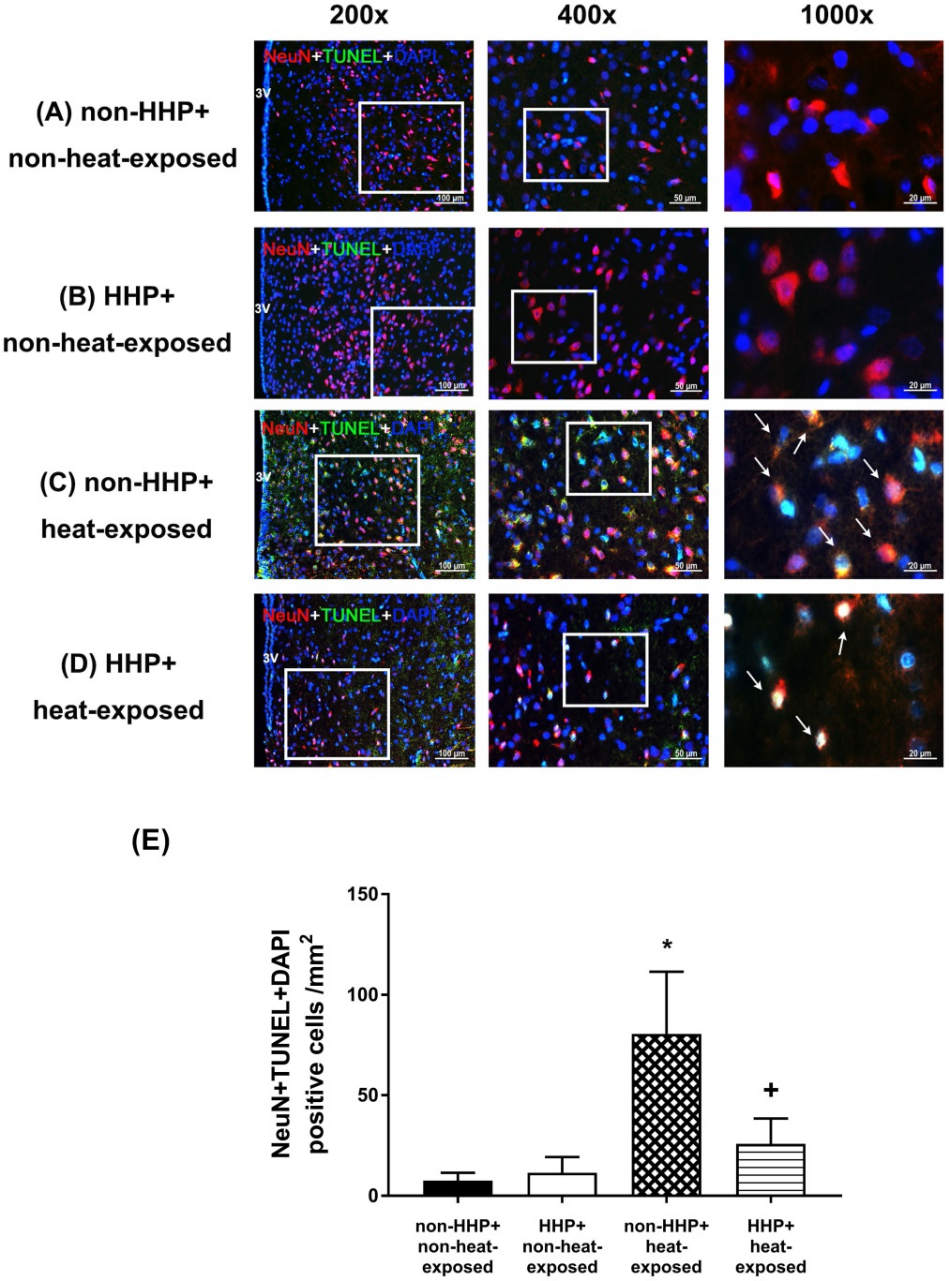
Photographs of immunofluorescence staining of hypothalamic apoptotic neurons (NeuN+TUNEL+DAPI-positive cells) in a non-HHP+non-heat-exposed rat (A), an HHP+non-heat-exposed rat (B), a non-HHP+ heat-exposed rat (C), and an HHP+ heat-exposed rat (D). The animals were killed 90 min after heat stress or at the equivalent time for the non-heat-exposed groups. Scale bar= 50 μm). (E) The values are presented as the mean±SDs; n=10 per group. *P<0.05 for the non-HHP+heat-exposed group versus the non-HHP+non-heat-exposed group; +*P*<0.05 for the HHP+heat-exposed group versus the non-HHP+heat-exposed group.

H&E staining revealed that the non-HHP + heat-exposed group exhibited significantly higher hypothalamic cell damage scores than the non-HHP + non-heat-exposed group and the HHP + non-heat-exposed group (0 vs. 2.5) (Fig. 3). Both the non-HHP+non-heat-exposed and the HHP+non-heat-exposed groups exhibited almost normal morphology, whereas in the non-HHP+heat-exposed group, ~50% of the field showed moderate damage (e.g., structural disorganization, edema, many pyknotic cells, and vacuolization). Although HHP did not significantly affect the morphology of the paraventricular region in the hypothalamus in non-heat-exposed rats, it significantly attenuated the increase in hypothalamic cell damage scores in heat-exposed rats (Fig. 3). Immunofluorescence staining further demonstrated that the non-HHP+heat-exposed rats exhibited significantly more NeuN+caspase-3+DAPI-positive cells (Fig. 4) and NeuN+TUNEL+DAPI-positive cells (Fig. 5) in the hypothalamus than the non-HHP+non-heat-exposed and HHP+non-heat-exposed rats. Although HHP did not affect the number of either NeuN+caspase-3+DAPI-positive cells (Fig. 4) or NeuN+TUNEL+DAPI-positive cells in the hypothalamus (Fig. 5), it significantly prevented the increase in the number of hypothalamic apoptotic neurons following heat exposure (Fig. 4 and Fig. 5).

### HHP increases the hypothalamic levels of HSP-72, HIF-1α, and HO-1 in heat-exposed rats

**Figure 6:**
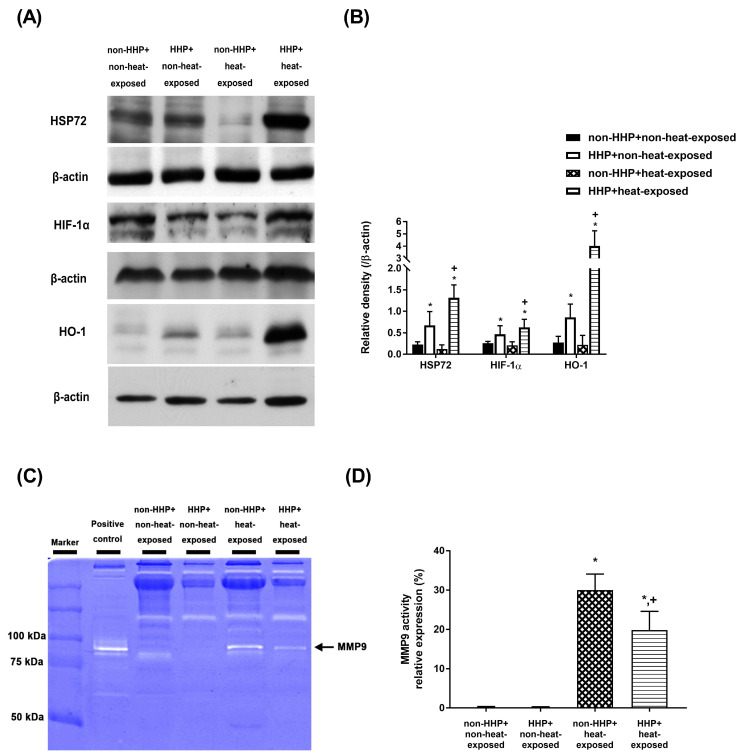
(A) Western blot analysis of HSP72, HIF-1α and HO-1 in hypothalamic tissues from a non-HHP + non-heat-exposed rat, an HHP + non-heat-exposed rat, a non-HHP + heat-exposed rat, and an HHP + heat-exposed rat. (B) The fold-change values represent the mean of 10 samples (n=10) divided by the mean of the 10 controls (n=10). (C) Gelatin zymography analysis of MMP-9 in hypothalamic tissues from a non-HHP + non-heat-exposed rat, an HHP + non-heat-exposed rat, a non-HHP + heat-exposed rat, and an HHP + heat-exposed rat. (D) The percent expression values represent the mean of 10 samples (n=10). The results are presented as the means±SDs *P<0.01 compared with the non-HHP and non-heat-exposed groups; ^+^*P*<0.01 compared with the non-HHP + heat-exposed group.

Western blot analysis revealed no significant differences in the hypothalamic levels of HSP-72, HIF-1α, and HO-1 between the non-HHP+non-heat-exposed group and the non-HHP+heat-exposed group (*P*>0.05; Fig. 6A and Fig. 6B). On the other hand, the HHP+heat-exposed rats exhibited significantly higher hypothalamic levels of HSP-72, HIF-1α, and HO-1 than the non-HHP+heat-exposed rats (*P*<0.05; Fig. 6A and Fig. 6B).

### HHP decreases heat-induced MMP-9 overexpression and activity in heat-exposed rats

Gelatin zymography analysis revealed that the non-HHP+heat-exposed group exhibited significantly higher levels of MMP-9 activity in the hypothalamus than the non-HHP+non-heat-exposed group and the HHP+non-heat-exposed group (Fig. 6C and Fig. 6D). However, the HHP+heat-exposed rats exhibited significantly lower hypothalamic MMP-9 activity than the non-HHP+heat-exposed rats (Fig. 6C and Fig. 6D).

## Discussion

In the present study, which used an experimental rat model of heatstroke, heatstroke-exposed animals displayed hyperthermia (i.e., Tco of 42 °C), arterial hypotension (i.e., MABP of 20 mmHg), bradycardia (i.e., HR of ~200 beats/min), hypothalamic ischemia (i.e., CBF of 40% of the original level), hypoxia (i.e., PO_2_ of ~5 mmHg), neuronal degeneration (i.e., increased neuronal damage scores) and apoptosis (i.e., increased numbers of Neu-N/caspase-3/DAPI-positive cells and NeuN+TUNEL+DAPI-positive cells). In addition, heat-induced hypothalamic ischemic/hypoxic injury were associated with increased hypothalamic levels of MMP-9 activity. The most striking finding of the present study was that HHP protected against heat-induced hypotension (e.g., increased MABP to ~60 mmHg), bradycardia (e.g., increased HR to ~350 beats/min), hypothalamic ischemia (e.g., increased CBF to ~100%), hypoxia (increased the PO_2_ to 17 mmHg), hypothalamic neuronal degeneration (e.g., decreased neuronal damage scores) and apoptosis (e.g., reduced the number of apoptotic neurons) and increased hypothalamic levels of MMP-9 activity. Simultaneously, HHP increased the hypothalamic expression of HSP-72, HIF-1α, and HO-1 in heat-exposed animals. Although HHP did not affect heat-induced hyperthermia, it attenuated heat-induced hypothalamic overexpression of MMP-9 as well as hypothalamic ischemia/hypoxia and its complications (e.g., hypothalamic neuronal degeneration and apoptosis).

HIF-1α maintains oxygen homeostasis and facilitates cellular adaptation to low oxygen conditions by regulating many downstream genes that participate in angiogenesis, erythropoiesis, energy metabolism, apoptosis and neuronal stem/progenitor cell proliferation 9. Heat acclimation (HA) confers protection against acute heat stress and delays thermal injury 29, 30. Increased levels of HIF-1α as well as HSP72 and its metabolic targeted genes are among the cytoprotective changes that occur during HA 31. HIF-1α, the master regulator of oxygen homeostasis, participates in the process of HA 32, 33. Our present data show that HHP exerts the same cytoprotective effects as HA. HHP may protect against heat-induced hypothalamic injury by upregulating HIF-1α. In contrast, our present results are not consistent with several previous findings. Neuron-specific HIF-1α-deficient mice display reductions in both the infarct volume and expression of proapoptotic genes following bilateral common carotid artery occlusion 34. In addition, early inhibition of HIF-1α with small interfering RNA reduces ischemia-reperfusion-induced brain injury in rats 35. Inhibition of HIF-1α by HIF-1α inhibitors such as 2-methoxyestradiol 35, mir-335 9 and hyperbaric oxygen 36 in the acute phase of ischemic stroke exerts beneficial effects via inhibition of apoptotic genes. Thus, downregulating HIF-α expression in the early period of cerebral ischemia could be efficacious in reducing cerebral ischemic damage. The reason for the discrepancy between our results and the results of these previous studies is currently unclear.

Transgenic mice have been used to examine the role of HSP72 in experimental heatstroke 6. Compared to transgene-negative littermate controls ([-]HSP72 mice), transgenic mice heterozygous for the porcine HSP-72 β gene ([+]HSP 72 mice) exhibit lower levels of cellular ischemia, inflammatory and oxidative damage markers in the hypothalamus and lower neuronal damage scores 4 hours after heat stress. [-]HSP72 mice were subjected to heat stress, and 4 hours after exposure, none of the 12 mice were still living, and the Tco of the mice was 34.2±0.4 °C. When [+]HSP-72 mice were exposed to the same heat treatment, the fraction survival and Tco values were significantly increased to 12/12 and 37.4±0.3 °C, respectively. This study indicates that HSP72 overexpression appears to be critical for the development of thermotolerance and protection from heat-induced hypothalamic ischemic and oxidative damage. In the present study, we further confirmed that HHP-induced HSP72 overexpression in the hypothalamus protects against ischemic/hypoxic injury in the hypothalamus in heat-exposed rats. In studies of cerebral ischemia, neurodegenerative disease, epilepsy, and trauma, HSP72 has been shown to be neuroprotective 37. Proteins that accumulate in Alzheimer's disease models are able to be degraded in HSP72-overexpressing transgenic mice, leading to neurological improvements 38.

Hemeoxygenase-1 (HO-1) is an inducible isoform of the first and rate-limiting enzyme of the degradation of heme into iron, carbon monoxide, and biliverdin. It possesses anti-inflammatory, antiapoptotic, angiogenic, and cytoprotective functions, suggesting that it plays potential protective and defensive role in several pathophysiological states, including ischemic stroke 39. HO-1 knockout mice exhibit greater ischemic damage than wild-type mice 40. Induction of HO-1 expression with acetyl-11-keto-β-boswellic acid 41 or saponins 42 effectively reduces stroke- or reperfusion-induced injury. These observations provide compelling information that HO-1 activation contributes to neuroprotection under many conditions. HO-1 exerts its anti-inflammatory effects by upregulating the expression of interleukin-10 (an anti-inflammatory cytokine) and interleukin-1 receptor antagonist 43. In the present study, HHP may have attenuated hypothalamic inflammation and ischemic/hypoxic injury by enhancing HO-1 expression in the hypothalamus.

MMP-9 plays several important roles associated with the actions of neutrophils, such as degradation of extracellular matrix, activation of IL-1β, and cleavage of several chemokines 44. In a mouse model, MMP-9 deficiency results in resistance to endotoxin shock, suggesting that MMP-9 is important in sepsis 45. Knockout of MMP 9 results in delayed apoptosis, vascularization, and ossification of hypertrophic chondrocytes 46. Enhanced MMP-9 expression and activity have been observed in many neuropsychiatric disorders and central nervous system disorders, including ischemic stroke 28, 47, 48. In cerebral ischemia, enhanced expression of MMP 9, which is associated with various complications, including excitotoxicity, neuronal damage, apoptosis, and BBB opening, which leads to cerebral edema and hemorrhagic transformation, is observed 7. It is evident that brain ischemia, inflammation, and oxidative damage rather than hyperthermia of the body are the main causes of heatstroke 1, 4. In addition, heatstroke resembles septic shock in many ways 1, 4. In the present study, HHP may have inhibited the overexpression of MMP-9 in the hypothalami of rats subjected to heatstroke, resulting in a reduction in hypothalamic ischemia, hypoxia, neuronal degeneration and apoptosis.

Hypoxia-inducible factors are key molecules that regulate the cellular response to hypoxia and inflammation and play a major role in normal cell function and survival 49. Current evidence also indicates that the HIF-1α pathway in the hypothalamus may be involved in the regulation of food intake and energy expenditure 49. Previous studies have shown that 30 cycles of intermittent hypoxia per hour for 8 hours per day for 21 days reduces body weight and BAT wet weight in rats 50. In our present study, we further confirmed that five hours of HHP (0.66 ATA or 18.3% O_2_) daily for 5 consecutive days per week for 2 weeks before the start of heat exposure caused both weight loss and reduced BAT wet weight in rats. BAT activation occurs at temperatures between 4 and16 °C 51. In our present study, all HHP experiments were conducted at 26 °C. It is, therefore, improbable that procedure-induced BAT behavior or uncoupling protein 1 function influenced the protective effects of HHP in heatstroke.

**Figure 7:**
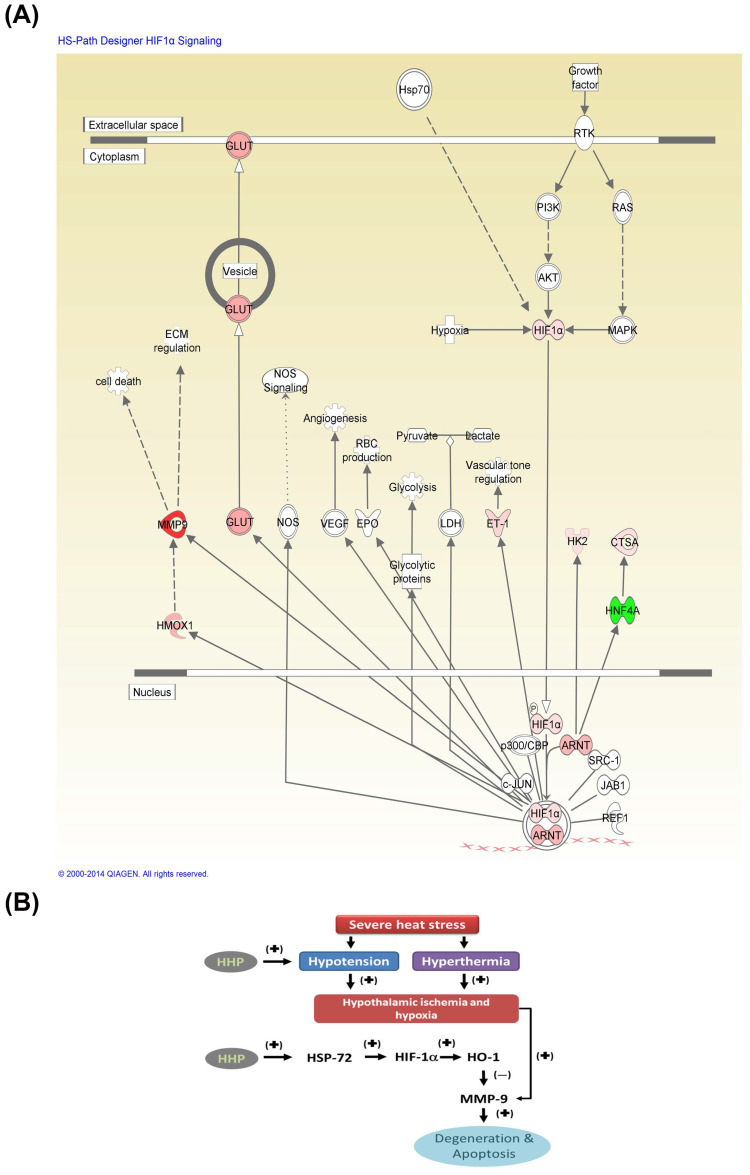
Core molecular net words identified by IPA. The altered genes are involved in biological functions associated with HIF-1α signaling (A). The red nodes and lines indicate the heat shock protein (HSP)70/HIF-1α/HMOX1 (or HO-1)/MMP-9 cell death pathway might be involved in the present results. (B) A flowchart of the proposed pathogenic mechanism of heatstroke in rats. Rodents subjected to heatstroke (evidenced by excessive hyperthermia and hypotension) exhibit higher levels of cellular ischemia and damage markers (e.g., MMP-9) in the hypothalamus, which leads to hypothalamic neuronal degeneration and apoptosis (I). Based on the core molecular net words identified by IPA shown in (B), HHP may attenuate heat-induced hypothalamic degeneration and apoptosis via the HSP70 (or HSP72)/HIF-1α/HO-1/MMP-9 pathways (II). (+), upregulation; (-) downregulation. HHP might ameliorate hypothalamic overexpression of MMP-9 in heat-exposed animals by reducing hypotension (or increasing CBF) and/or by enhancing HSP-72/HIF-1α/HO-1 signaling.

Fig. 7A depicts core the molecular networks identified by ingenuity pathway analysis (IPA) 5. The altered proteins, including HSP72, HIF-1α, HO-1, MMP-9, and other ischemia-related mediators, are involved in significant biological functions associated with HIF-1α. Severe heat stress may cause both hypotension and hyperthermia and result in hypothalamic ischemic/hypoxia and overexpression of MMP-9, leading to hypothalamic degeneration and apoptosis (Fig. 7B). HHP may attenuate heat-induced hypothalamic degeneration and apoptosis by inhibiting the pathways involved in hypotension, hypothalamic ischemic and hypothalamic MMP-9 overexpression or enhancing the HSP-72/HIF-α/HO-1/MMP-9 pathway.

## Limitations

This study used adult male rats and therefore cannot reflect the complicated human clinical situation, which may involve different comorbidities. Male rats were used to avoid the hormonal fluctuations that occur in female rats. Additionally, in this study, thermal experiments were performed in rats under general anesthesia.

## Conclusions

Based on our present data, we conclude that severe heat stress causes hypothalamic ischemia/hypoxia, upregulation of hypothalamic MMP-9, and hypothalamic neuronal degeneration and apoptosis. HHP upregulates the hypothalamic protein expression of HSP-72, HIF-1α, and HO-1 and downregulates hypothalamic MMP-9 activity to attenuate hypothalamic neuronal degeneration and apoptosis in heat-exposed animals.

## Figures and Tables

**Table 1:** Means and standard deviations (SDs) of body weight, BAT weight and BAT temperature in the two experimental groups (n=6 per group)

	Non-HHP	HHP
Initial body weight (g)	274±44	275±45
Final body weight (g)	277±42	255±43
Initial - final weight (△weight; g)	30±6	-17±7*
BAT weight (g)	0.59±0.08	0.42±0.06*
BAT temperature (°C)	35.8±0.02	35.5±0.03

*p<0.05, HHP vs. non-HHP; HHP, hypobaric hypoxic preconditioning; BAT, brown adipose tissue.

## Abbreviations

HIF-1α: Hypoxia-inducible factor-1α
HSP-72: Heat shock protein-72
HO-1: Hemeoxygenase-1
MMP-9: Matrix metalloproteinase-9
HHP: hypobaric hypoxia preconditioning
CBF: cerebral blood flow
PO_2_: partial oxygen pressure
Ta: ambient temperature
Tco: colon temperature
MABP: mean arterial blood pressure
HR: heart rate
NeuN: neuronal nuclei
Caspase-3: Cysteine-aspartic acid protease 3
DAPI: 4',6-Diamidino-2-phenylindole
IPA: ingenuity pathway analysis
BBB: blood-brain barrier
3V: third ventricle
ME: median eminence
